# Identification of an early-stage Parkinson’s disease neuromarker using event-related potentials, brain network analytics and machine-learning

**DOI:** 10.1371/journal.pone.0261947

**Published:** 2022-01-07

**Authors:** Sharon Hassin-Baer, Oren S. Cohen, Simon Israeli-Korn, Gilad Yahalom, Sandra Benizri, Daniel Sand, Gil Issachar, Amir B. Geva, Revital Shani-Hershkovich, Ziv Peremen

**Affiliations:** 1 Movement Disorders Institute and Department of Neurology, Chaim Sheba Medical Center, Tel Hashomer, Ramat-Gan, Israel; 2 Sackler Faculty of Medicine, Tel-Aviv University, Tel-Aviv, Israel; 3 Department of Neurology, Assaf Harofeh Medical Center, Zerifin, Israel; 4 Department of Neurology and Movement Disorders Clinic, Shaare Zedek Medical Center, Jerusalem, Israel; 5 Movement Disorders Unit, Functional Neurosurgery Center, Assuta Ramat Ha Hayal Hospital, Tel Aviv, Israel; 6 elminda Ltd., Herzliya, Israel; 7 Faculty of Medicine, Department of Medical Neurobiology, The Hebrew University of Jerusalem, Ein Kerem, Jerusalem, Israel; 8 Department of Electrical and Computer Engineering, Ben-Gurion University of the Negev, Beer-Sheva, Israel; National University of Sciences and Technology, PAKISTAN

## Abstract

**Objective:**

The purpose of this study is to explore the possibility of developing a biomarker that can discriminate early-stage Parkinson’s disease from healthy brain function using electroencephalography (EEG) event-related potentials (ERPs) in combination with Brain Network Analytics (BNA) technology and machine learning (ML) algorithms.

**Background:**

Currently, diagnosis of PD depends mainly on motor signs and symptoms. However, there is need for biomarkers that detect PD at an earlier stage to allow intervention and monitoring of potential disease-modifying therapies. Cognitive impairment may appear before motor symptoms, and it tends to worsen with disease progression. While ERPs obtained during cognitive tasks performance represent processing stages of cognitive brain functions, they have not yet been established as sensitive or specific markers for early-stage PD.

**Methods:**

Nineteen PD patients (disease duration of ≤2 years) and 30 healthy controls (HC) underwent EEG recording while performing visual Go/No-Go and auditory Oddball cognitive tasks. ERPs were analyzed by the BNA technology, and a ML algorithm identified a combination of features that distinguish early PD from HC. We used a logistic regression classifier with a 10-fold cross-validation.

**Results:**

The ML algorithm identified a neuromarker comprising 15 BNA features that discriminated early PD patients from HC. The area-under-the-curve of the receiver-operating characteristic curve was 0.79. Sensitivity and specificity were 0.74 and 0.73, respectively. The five most important features could be classified into three cognitive functions: early sensory processing (P50 amplitude, N100 latency), filtering of information (P200 amplitude and topographic similarity), and response-locked activity (P-200 topographic similarity preceding the motor response in the visual Go/No-Go task).

**Conclusions:**

This pilot study found that BNA can identify patients with early PD using an advanced analysis of ERPs. These results need to be validated in a larger PD patient sample and assessed for people with premotor phase of PD.

## Introduction

Neurodegenerative diseases are major causes of physical and cognitive dysfunction leading to declining function and quality of life in older people. Disease-modifying therapy (DMT) is not yet available for these disorders, but when developed, it should be administered early in the neurodegenerative process to minimize irreversible accumulative brain damage, preferably during the prodromal or preclinical phases to maximize their effect [1–4]. A major barrier to DMT development is the lack of effective tools for early diagnosis and for objective monitoring of disease activity during clinical trials. In Parkinson’s disease (PD), CSF- or blood-based biomarkers have not yet been proven specific enough for clinical utility for diagnosis or longitudinal monitoring [5]. Despite recent advances, imaging methods used in PD are based mainly on detecting degeneration of motor pathways [6]. However, the neuropathological process in PD is known to begin many years before the onset of motor symptoms, beginning in extra-nigral locations, including the lower brainstem, olfactory bulb, and the peripheral autonomic nervous system [5].

Among the non-motor symptoms of PD, cognitive impairment is highly prevalent and while it can occur in a subtle form as mild cognitive impairment (MCI) in the early stages in up to 25% of newly diagnosed patients [7,8], it tends to worsen and become very disabling [9]. The decline in cognitive function affects several domains including attention, working memory and executive functions, language, visuospatial skills, and episodic memory. Researchers have suggested to divide the impairments into two distinct syndromes based on their underlying brain structures and neurotransmitter involved, known as the ‘dual syndrome hypothesis’ [10]. The first syndrome arises at early stages, is related to dopamine depletion in the basal ganglia (BG) and causes disruption of the cortico-basal ganglia-thalamo-cortical (CBGTC) loops [11,12]. These are parallel and segregated channels [13] of sensorimotor, associative, and limbic information projecting from the cortex to the BG, and back to the cortex through the thalamus [12–14]. Within these, the motor loop includes a direct pathway, where dopamine is thought to excite neurons and result in activation of motor cortex, and an indirect pathway where dopamine inhibits neuronal activity resulting in no motor output [11,15,16]. In this respect, the BG plays a modulatory role in cognition and action by modulating cognitive processes in the cortex such as reinforcement learning and action selection via the direct pathway [11], and inhibitory control via the indirect pathway [17]. Aberrant neural activity in the associative or limbic streams may lead to further cognitive and behavioral symptoms in PD patients [12]. Deficits in executive functions such as attention, working memory, planning and response inhibition were demonstrated to involve the BG structures in studies using electrophysiological recordings from implanted electrodes in PD patients [16–19], some of which used simultaneous recording from the BG and cortex, including surface electroencephalography (EEG) recording [16,17,19]. Further evidence to the role of BG in cognitive function comes from functional magnetic resonance imaging (fMRI) studies [20,21]. The second PD cognitive syndrome occurs in later stages of the disease, is related to the posterior cortex and temporal lobe degeneration and is associated with cholinergic deficiency; it manifests mainly with visuospatial memory deficits and eventually leads to dementia [9,10].

Functional imaging methods, including resting-state fMRI and metabolic brain imaging such as Fluorine 18-labelled-fluorodeoxyglucose-positron emission tomography have identified disease-specific network signatures in PD patients, which have been proposed as potential markers in early-stage PD (ESPD) [22,23]. However, it may be technically as well as economically more practical to use high-resolution EEG to identify neurophysiological fingerprints of ESPD. Furthermore, EEG is non-invasive and does not require the use of radionuclides. Indeed, prior studies have shown abnormal brain activity in ESPD patients based on resting-state EEG [24–29].

In recent years, machine learning (ML) and deep learning algorithms have been used to identify EEG-based neuromarkers in PD, including early-stage markers [30–35]. These efforts relied mainly on resting-state EEG. However, event-related potentials (ERPs), occurring in response to a specific stimulus during a cognitive task, reflect perceptual and cognitive functions, and the integrity of their underlying neuronal networks. Abnormal ERPs can be observed in individuals with neurological conditions in which brain circuitry is impaired, and consequently show cognitive dysfunction. In line with this, ERP measures have yielded various electrophysiological markers representing cognitive impairment in PD (reviewed in [36,37]).

The Go/No-Go and Oddball tasks involve cognitive functions related to the earlier, executive function syndrome of PD, which rely on the frontal cortex. The Go/No-Go task mainly assesses response inhibition (reviewed in [38]). The Oddball task is commonly used for assessing attention and cognitive function including working memory [39]. Both tasks have been tested in PD patients [36,37,40,41].

In the current study, we obtained high-resolution EEG recordings during performance of the visual Go/No-Go (VGNG) and auditory Oddball (AOB) cognitive tasks from individuals with early-stage and later-stage PD, and from healthy controls (HC). ERPs were processed using a novel technology known as Brain Network Analytics (BNA) [42–44] to generate representations of brain activity. The BNA representations were then analyzed by a ML algorithm with the goal of identifying candidate neuromarkers of ESPD. We hypothesized that ESPD patients would manifest cognitive deficits elicited in the chosen tasks, arising from dysfunction of the CBGTC circuit, which would be captured by surface EEG. Furthermore, we hypothesized that combining these single features in a data-driven approach by ML tools would provide a more accurate ESPD neuromarker.

## Methods

### Study participants

Eligible PD patients and HC were right-handed individuals between ages 40 and 80 years. Participants in the PD group were diagnosed as idiopathic PD according to the UK Brain Bank Criteria [45]. PD participants at regular follow-up at the Movement Disorders Institute at the Chaim Sheba Medical Center, Ramat Gan, Israel were included. Some were taking part in a repetitive transcranial magnetic stimulation (rTMS) study with brain monitoring; however, the current analysis used only data obtained before rTMS treatment. HC were recruited through advertisement in Sheba Medical Center. Exclusion criteria were: 1) an unstable medical condition, or 2) a history of epilepsy, dementia, cerebrovascular disease, previous head injury, brain tumor, or any craniosurgical intervention; 3) active depressive or psychotic symptoms, current drug abuse or alcoholism, antipsychotic treatment. Eligible participants had to score below 14 on the Beck Depression Inventory (BDI) [46] and above 25 on the mini-mental state exam (MMSE) [47] or above 23 on the Montreal Cognitive Assessment (MoCA) [48]. The study was approved by the institutional Helsinki committee and all participants gave their written informed consent.

PD participants on stable anti-Parkinsonian therapy for at least one month before enrollment were subdivided into 2 groups. The first, ESPD, that formed the neuromarker identification group, consisting of patients who had been diagnosed for 2 years or less, were on Hoehn and Yahr stage 1 or 2 [49] and could be on medical treatment for PD except for Levodopa. A second group, established PD, included patients who had been diagnosed for over 2 years and could be treated also with Levodopa. Data from this group were used for further exploration of the neuromarker.

### Study procedures

All study procedures were performed at the Movement Disorders Institute. For the current study, each participant visited the site on 2 or 3 occasions within a period of 2 weeks: once for clinical evaluations and twice for EEG recording (in case of 2 visits, clinical evaluation was performed on the first visit). In the current analysis, only data from the first EEG recording visit were used.

### Clinical assessments

All PD patients were evaluated according to the Hoehn and Yahr staging scale and the motor examination (part III) of the Unified Parkinson’s Disease Rating Scale (UPDRS) [50] in the off-medication condition. Additional clinical evaluations included the MMSE or the MoCA, and the BDI. For cognitive screening, PD patients were administered the MMSE or the MoCA, while HC completed only the MMSE. For analysis, we used a conversion from MoCA to MMSE following Roalf and colleagues [51,52].

### Stimuli and experimental paradigm

EEG recording was conducted in the off-medication condition, while participants performed the AOB and VGNG cognitive tasks (participants in the rTMS study completed 3 additional tasks not reported here). The AOB task included a series of 400 tones, 80% frequent (2000 Hz), 10% target (1000 Hz) and 10% novel complex sounds, which varied across trials (S1A Fig). Sounds were presented at a rate of 1 every 1.5 sec at 70 dB SPL. Each task contained two blocks of 200 trials separated by a 1 minute break and took approximately 15 minutes to complete. Subjects were required to respond to the target tone by pressing a key. During the VGNG task, letters were presented for 150 ms, either target or non-target (white on black background, height of 1.64 degrees, flanked by white vertical lines which remained constant on the screen throughout the block). The next letter was presented after a blank period of 850–2450 ms. There were 400 trials in total. Participants were instructed to press the button with their right index finger as quickly and accurately as possible in response to the frequent target letters (B, C, D, E, F, G) presented in the center of the screen (the Go condition; 80% of all trials) and not to respond to the rare non-target letter ‘X’ (the No-Go condition; 20% of all trials) (S1B Fig). For both tasks, there was a short practice period before EEG recording started. Only epochs with correct responses were analyzed.

### EEG recordings

Participants were seated in a quiet room, at a distance of 70 cm from a 19-inch LCD monitor. They were instructed to avoid eye and body movements as much as possible and maintain their gaze at the center of the screen during task performance. Clinical evaluation and EEG recording took approximately 3 hours to complete. High-density EEG was recorded using the 64 Ag-AgCl electrodes BrainCap TMS with Multitrodes system (BrainProducts, Gilching, Germany). The reference electrode was FCz, and data were sampled at 250 Hz.

### EEG processing

The EEG signals were recorded and cleaned as previously described [42]. Briefly, the EEG was referenced to average mastoid electrodes, and signals were band-pass filtered into overlapping physiological frequency bands of delta (0.5–4 Hz), theta (3–8 Hz), alpha (7–13 Hz) and beta (12–30 Hz). Then the recordings were cut into epochs based on stimulus onset and response, and averaged across trials of the same condition, producing ERPs. For the VGNG data, epoch segments (200 ms pre-stimulus to 800 ms post-stimulus) were averaged separately for the “Go” and the “No-Go” stimuli. In addition, motor-related activity was assessed by analyzing response-locked ERPs. Epoch segments (400 ms pre-response to 500 ms post-response) were averaged for the response-locked Go condition. For the AOB data, epoch segments (200 ms pre-stimulus to 1200 ms post-stimulus) were averaged separately for the “Frequent”, “Novel” and “Target” sounds. These ERPs were used as input data for the BNA analysis.

### Brain Network Analytics (BNA)

The BNA methods have been previously described [42]. Briefly, BNA produces a set of event-related spatiotemporal parcels (hereafter referred to as ‘STEPs’) of the ERP activity. Each STEP contains information about the magnitude, temporal and spatial features of the ERP including amplitude, latency, spatial location (left-right and posterior-anterior, each on a scale of -1 to 1) and a topographic similarity score (S2 Fig). After STEPs are generated for each participant in each frequency band of the ERP, clustering is applied to generate group STEPs that represent spatiotemporal events common to at least 70% of the subjects in the group. A pool of healthy subjects divided into age bins (120 subjects per bin) was used to generate each STEP. For individual participants, STEPs corresponding to group STEPs in a matching process are selected and their attributes are calculated (i.e., BNA scores). The topographic similarity scores represent the similarity of the participant’s STEP to the normative group’s STEP in terms of shape and dynamics around the peak, after spatially and temporally aligning the peaks. STEP attributes served as input data for the ML algorithm as discussed below. In addition to the BNA scores, the ERP variability (ERPv) was calculated. This measure considers the variability of the ERP waveform (at a frequency band of 0.5–30 Hz) across multiple trials in a single electrode and is based on the standard error (SE). Low ERPv reflects good repeatability across multiple trials, while high ERPv reflects the variability of the neural response to the same stimulus. The ERPv is averaged across electrodes in each of nine scalp regions as detailed in S2 Table.

### Machine-learning based classification

**Fig 1:**
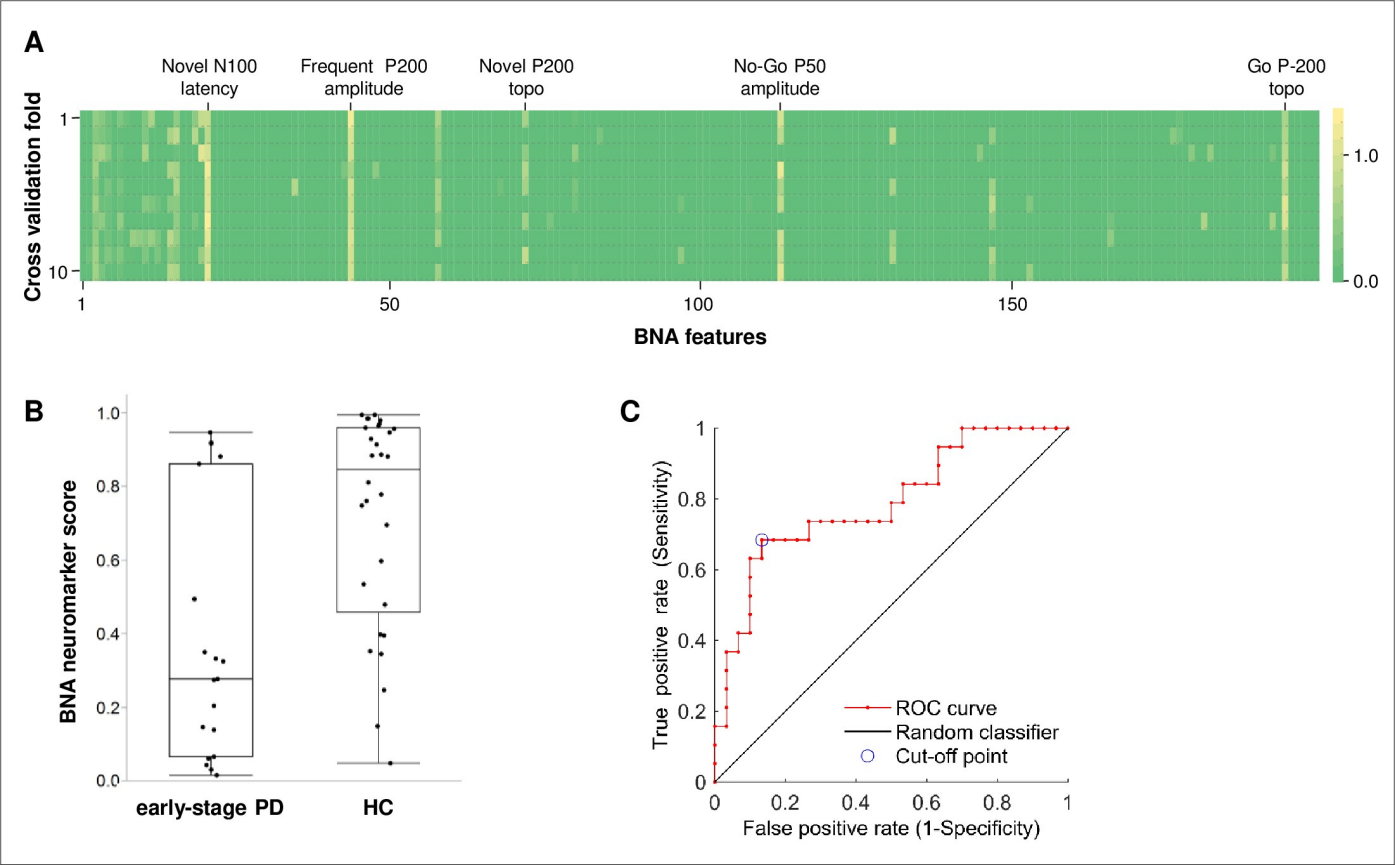
The BNA neuromarker characteristics. (A) Weights of each feature in each iteration (fold) of the cross-validation process. The consistency of the selected features in each fold can be observed. The names of the 5 most important features of the final model are indicated at the top. (B) BNA neuromarker scores for participants in the ESPD and the HC groups. The scores were obtained from the cross-validation process and represent [1- probability for ESPD]. Whiskers extend to the highest and lowest observations, boxes represent 25–75 interquartile ranges, and horizontal bars represent medians. Mann-Whitney U test (normal approximation): U = 120, Z = -3.38, p-value = 0.0007. (C) Receiver-operating characteristic (ROC) curve for the BNA neuromarker. The cross-validation scores were used for the ROC analysis. topo, topographic similarity; PD, Parkinson’s disease; HC, healthy controls.

We used 199 engineered features extracted by the BNA analysis from the HC and ESPD groups as input data for the ML model training to identify a neuromarker associated with ESPD. To lessen over-fitting, we used the False Positive Rate (FPR) feature selection method to reduce the number of input features for training a logistic regression (LR) classifier. The LR classifier predicted the probability of being healthy (i.e., [1- probability for ESPD]). The classification model was evaluated with a 10-fold cross-validation stratified by group (in each iteration, data of 3 HC and 2 ESPD were left out). In each iteration, the FPR feature selection method was applied, followed by the LR classifier based on the selected features. Hence, each cross-validation iteration resulted in a model, which coefficients were used to calculate the scores for the left-out subjects (i.e., cross-validation scores). The scores from the cross-validation iterations were also used for the ROC analysis. Finally, we ran feature selection and fitted a model on the full data set of the HC and ESPD groups, to get the final model. The coefficients of the final model were used to calculate the linear term for the established PD group (see below). Fig 1A shows the weight of each feature in each iteration (i.e., fold). The weight of each feature in the final model was used to determine its importance. In the logistic function:

$$p=\frac{1}{1+e^{-(b_{0}+b_{1}x_{1}+b_{2}x_{2}+\cdots +b_{m}x_{m})}}$$ (1)

*b*_i_ is the coefficient for BNA feature i; *x*_i_ is an observation value for BNA feature i. The linear term, marked in grey in Eq (1), is a weighted sum of the input features.

As an exploratory analysis, the relevance of the BNA score to PD symptoms was assessed by correlating the outcome of the linear term of the final LR model with the motor UPDRS (mUPDRS) scores from the established PD group. We took the linear term values rather than the probability values for checking the relevancy of the model to PD symptoms, since we used a linear correlation analysis and considering the non-linear nature of the logit function. It is important to note that the data of the established PD group were not part of the training set.

### Statistical analysis

Statistical analyses were carried out using JMP version 12 software (SAS Institute Inc., Cary, NC, 1989–2019). The majority of the demographic, clinical and task performance measures were not normally distributed and therefore they were compared between groups by the non-parametric Wilcoxon rank-sum test or a Chi-square test. P-values <0.05 were considered statistically significant. Performance measures were adjusted for multiple comparisons with Bonferroni correction. Response time was measured using the median response time of correct responses. Correlations were assessed using Pearson’s correlation coefficient.

## Results

Thirty HC participants and 21 participants with early-stage idiopathic PD were enrolled in the study. All participants completed the cognitive tasks in each of two EEG recording sessions. Two ESPD participants had poor quality data and are not included in the analysis, leaving 19 participants in the ESPD group. Demographic and relevant clinical characteristics of study subjects are shown in Table 1. The percentage of male participants and BDI scores (a trend) were higher, while the average MMSE scores were lower in the ESPD group in comparison to the HC group.

**Table 1:** Demographic and clinical characteristics of participants included in the BNA neuromarker.

	Early-stage PD	Healthy Control	P-value
Number of participants	19	30	
Males, n (%)^a^	16 (84)	15 (50)	0.012
Mean age, years (SD)	63.7 (7.8)	64.4 (6.2)	NS
Mean duration of PD, years (SD)	1.1 (0.9)	-	
Mean Hoehn and Yahr stage (SD)	1.8 (0.6)	-	
Mean BDI score (SD)	5.4 (3.7)	3.5 (3.7)	0.059
Mean MMSE score (SD)	26.2 (2.1)	29.7 (0.8)	<0.001
Mean mUPDRS score (SD)	20.1 (8.8)	-	
Treatments for PD, number of patients (%)^b^			
Levodopa	0		
Monoamine oxidase B inhibitor	15 (79)		
Dopamine agonist	5 (26)		
Amantadine	7 (37)		
Anticholinergic agent	1 (5)		

^a^Chi-square test.

^b^Patients can be on multiple treatments.

SD, standard deviation; BDI, Beck Depression Inventory; MMSE, mini mental status scale; mUPDRS, motor examination (part III) score of the Unified Parkinson’s Disease Rating Scale.

### Cognitive task performance

Behavioral results of the VGNG and AOB tasks in the ESPD and HC groups, are shown in Table 2. One ESPD patient performed the AOB below chance level and was therefore not included in the AOB performance analysis. There were no significant differences in the AOB task performance between groups. In the VGNG task, accuracy was significantly lower in the ESPD group than in the HC group.

**Table 2:** Results of the cognitive tasks performance.

	Early-stage PD	Healthy Control	P value
**Visual Go/No-Go task**			
Number of participants	19	30	
Response time, ms	420.3 (56.9)	421.9 (47.0)	NS
Response time SD, ms	92.07 (21.5)	90.8 (17.6)	NS
Accuracy, %	92.4 (4.1)	95.4 (2.7)	0.005*
Omission errors, %	4.1 (4.9)	1.6 (2.4)	NS
Commission errors, %	21.4 (14.0)	16.2 (9.9)	NS
**Auditory Oddball task**			
Number of participants	18	30	
Response time, ms	474.9 (88.0)	504.5 (85.8)	NS
Response time SD, ms	101.9 (35.0)	106.1 (33.0)	NS
Accuracy, %	99.4 (0.5)	99.1 (1.9)	NS
Omission errors, %	3.5 (4.0)	2.5 (3.5)	NS
Commission errors, %	0.27 (0.4)	0.64 (2.0)	NS

*Statistically significant after Bonferroni correction for multiple comparisons.

Performance values are mean (±standard deviation) per group; ms, milliseconds; NS, not significant.

### Discriminant ability of the BNA neuromarker

The output of the BNA system was analyzed by the ML algorithm (see Methods and Fig 1A), which identified a BNA neuromarker composed of 15 features. Box plots with the neuromarker scores from the cross-validation process for the ESPD and HC groups are shown in Fig 1B. The cross-validation scores are more representative in terms of model validity, in the case a validation set is lacking.

The discriminant ability of the BNA neuromarker was assessed using a receiver-operating characteristic (ROC) analysis (Fig 1C). Again, the cross-validation scores were used for the ROC analysis. The area under the ROC curve was 0.79 (95% CI: 0.65, 0.93). At the cutoff point that maximizes sensitivity while specificity is still above chance level, sensitivity was 74% and specificity was 73%. The ROC curve data in S3 Table shows various sensitivity-specificity values for different cutoffs.

While the control’s gender was balanced, the ESPD group was predominantly male (84%). This could potentially lead to a false neuromarker identification due to gender differences in the groups rather than disease-related difference. To check this possibility, we first examined the difference between the neuromarker scores of males and females in the HC group, which is gender balanced. A Wilcoxon rank-sum test showed no statistically significant effect (Z = -0.83, p-value = 0.41). Second, we ran 5 iterations of the ROC analysis with balanced gender across groups, i.e., taking each time data of randomly chosen three females (with no repeat) from the HC group and all the 15 HC males. The average area under the curve was 0.77 (range 0.76–0.78). At the cutoff point that maximizes sensitivity, while specificity is maximal above chance level for this sensitivity value, sensitivity was 0.74 (in all iterations) and average specificity was 0.69 (range 0.67–0.72). These results are very similar to those obtained with all HC females and thus suggest that gender differences are not the basis of the neuromarker discrimination between patients and HC.

### Components of the BNA neuromarker

**Fig 2:**
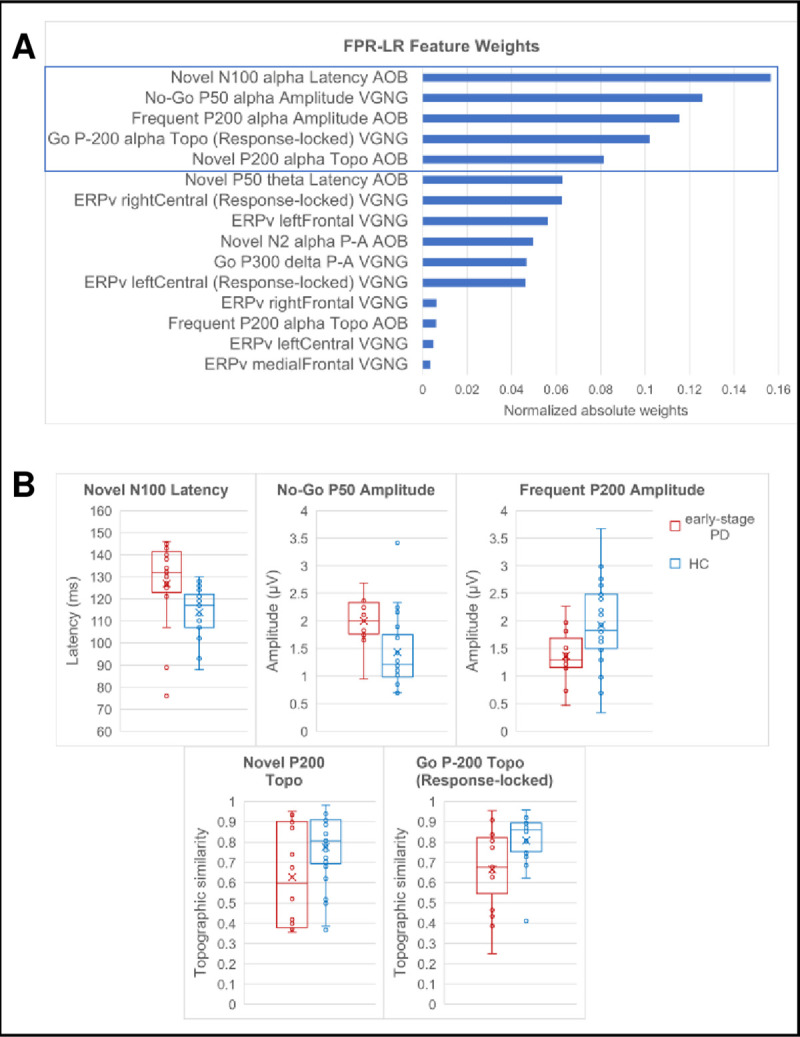
Features of the BNA neuromarker. (A) Weights of the BNA neuromarker features. The box indicates the five most important features. Weights are absolute value model weights normalized by their sum. (B) Box plots of the BNA scores of the five most important neuromarker features in participants with ESPD and HC. See Fig 1 for boxplot description. Points outside the whiskers are outliers, exceeding 1.5 times the interquartile range below the 1st quartile or above the 3rd quartile. Values are provided in S1 Table. AOB, auditory Oddball; VGNG, visual Go/No-Go; Topo, topographic similarity; ERPv, event related potential variability; μV, micro volts; ms, milliseconds.

The weights of the 15 features of the BNA neuromarker are shown in Fig 2A. The most important features, as determined by their weights, included the P50 amplitude from the No-Go condition and the N100 latency from the Novel condition, both of which are associated with early sensory processing; the P200 amplitude from the Frequent condition and the P200 topographic similarity from the Novel condition, associated with filtering of information; and P-200 topographic similarity from the response-locked Go condition, activity which precedes the motor response and is suggestive of reflecting motor-related processes. These features were ERPs in the alpha frequency band. Other features of the BNA neuromarker were ERPv scores of frontal and central locations from the response-locked and stimulus-locked VGNG ERPs. Two other components with relatively modest contributions to the BNA neuromarker were related to cognitive control and novelty processing (Novel N2 P-A in alpha band) [53] and Go processing including response preparation and execution [54] (Go P300 P-A in delta band; a “target” in the sense that it requires a response) [55]. See S4 Table for a summary of physiological explanations of the features.

The differences between the ESPD and the HC groups, in the neuromarker features with the highest importance, are shown in Fig 2B. Compared with HC, ESPD patients had higher No-Go P50 amplitude, slower Novel N100 latency, lower Frequent P200 amplitude, and lower Novel P200 and response-locked Go P-200 topographic similarity.

### Further exploration of the neuromarker

**Fig 3:**
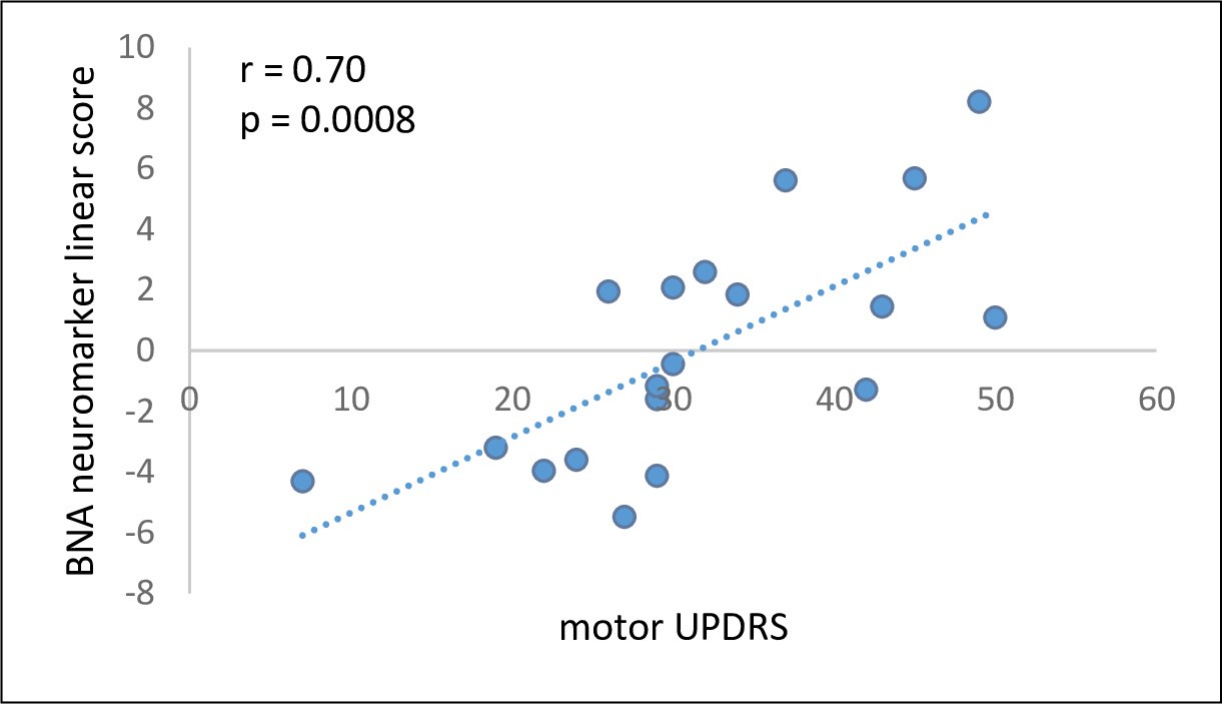
Correlation between the BNA neuromarker linear scores and the mUPDRS scores. Correlation between the BNA neuromarker linear scores and the mUPDRS scores in an independent established PD group (N = 19; one subject was assessed only in the on-state and was not included). mUPDRS, the motor examination (part III) of the Unified Parkinson’s Disease Rating Scale.

Twenty established PD patients went through the same procedures as the ESPD patients (see S5 Table for demographic and relevant clinical characteristics). The established PD patients’ scores from the linear term of the LR model (see Methods) correlated significantly with their mUPDRS scores (r = 0.70, p = 0.0008, 95% CI: 0.36, 0.88; Fig 3).

**Fig 4:**
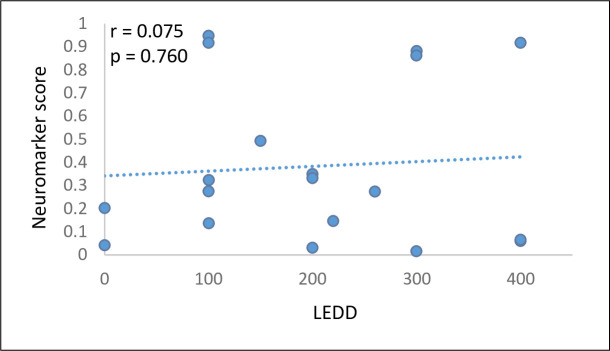
BNA Neuromarker scores as a function of LEDD. LEDD, levodopa equivalent daily dose.

Lastly, we calculated for each early-stage patient the levodopa equivalent daily dose (LEDD), a measure of the total daily medication a patient receives, which enables comparison across patients with different drug regimens [56]. LEDD was then correlated with the neuromarker score. As can be seen in Fig 4, there was no correlation between the neuromarker and the LEDD (r = 0.075, p = 0.760, 95% CI: -0.39, 0.51).

## Discussion

In this exploratory study we used BNA to analyze ERPs from ESPD patients and HC to establish a BNA neuromarker for ESPD. Using a ML algorithm, we identified a neuromarker composed of 15 BNA features with an AUC of 0.79 on ROC analysis and with sensitivity of 68% and specificity of 87%.

Notable in the current study, ERP signals were sensitive for discriminating ESPD patients from HC, while behavioral results differed only in accuracy of the VGNG task between groups. This result shows that electrophysiological ERPs capture deviations in specific cognitive processing aspects at early stages of the disease that are less manifested on the coarser behavioral level, which may benefit from compensatory mechanisms.

The BNA features with the highest importance could be classified into three cognitive functions: early sensory processing (P50, N100) [57], filtering of information (also defined as withdrawal of attention from irrelevant information; P200) [58] and motor-related processes (response locked P-200) [59,60]. Previous studies have shown alterations in the P50, N100 and P200 ERP components in PD patients, although some studies failed to find differences from HC and other showed controversial results (for an exhustive review see [61]). Many ERP studies have shown altered motor-related potentials in PD, mainly in self-initiated movements [62]. However, alterations in motor-related potentials of externally cued movements, which are more similar to our task-derived response-locked ERPs, were also found [63,64].

The present analysis is unique and differs from previous ERP studies in the BNA scores that entered feature selection. These included early and late ERP components from each and every task condition, in addition to introducing new features such as topographic similarity and posterior-anterior measures of ERPs, ERPv and ERPs in various frequency bands. In contrast, earlier research analyzed only a few commonly studied ERPs from each task condition. Thus, we enabled selection of features that previously were not subject to analysis. In addition, the majority of previous work showed ERP alterations in established PD rather than in ESPD patients. Our criteria for early stage, ≤2 years, were more stringent as compared to previous EEG studies of ESPD [25,26].

Previous studies showed differences between PD patients and HC in the AOB task, mainly in the P300 ERP components, which include P3a and P3b. The amplitude of the P3a component elicited by the Novel stimuli, which is thought to be mediated by dopaminergic activity [65] and to reflect attention allocation towards potentially important changes in the environment, is attenuated in PD (reviewed in [36,37]). The P3b component elicited by the Target stimuli, reflects updating of working memory representations in the prefrontal cortex [39,65]. Surprisingly, our neuromarker did not include P300 features of the AOB Novel or Target stimuli. Only the VGNG Go P300 was included in the neuromarker with a relatively moderate weight. Although the Go P300 is a “target” in the sense that it requires a response, its frequent occurrence in the task (80%) makes it different from the rare AOB Target stimulus. It might be that only in later stages of PD, the AOB P3a and P3b components are aberrant enough to hold a significant difference from HC.

ESPD subjects were on stable anti-parkinsonian therapy, which might raise concerns that we found a neuromarker of the therapy rather than a neuromarker of the disease. However, we used several means to limit drug effects. The first was excluding levodopa and anti-cholinergic drugs for the early-stage patients, agents known to induce EEG changes [66,67] (there was one patient exception for anticholinergic drug). Second, patients were tested in the off-medication state, in the morning, before receiving their drugs. Lastly, there was no correlation between the neuromarker score and LEDD, showing that the neuromarker does not represent drug effects.

As the study sample size was not large enough to have an independent validation group, we used data from an established PD patient group (disease duration > 2 years, N = 20) for evaluating the relevance of the neuromarker to other PD symptoms. The scores from the linear term of the LR model correlated significantly with the mUPDRS scores suggesting that the BNA neuromarker captures some aspects related to the motor symptoms severity, the hallmark of PD.

Several ML-based classification models for ESPD diagnosis have been reported (reviewed in [30]), which showed higher sensitivity and specificity than the current findings. However, most of these implied expensive, invasive, or complicated medical assessments. In contrast, the BNA neuromarker is based on the accessible and available EEG acquisition, which is optimal for use in a clinical setting and can be easily repeated over time for monitoring purposes.

The major limitations of the current study include the small study sample size and the lack of a validation group. Additionally, the difference in gender distribution and baseline MMSE scores between groups may present confounders affecting the results. It is important to note that the greater proportion of PD males than females in our study, reflects the higher (1.5–2 times) incidence and prevalence of PD in men than in women in the general population [68,69]. Taken together, it is of true concern that the biomarker is a consequence of a combination of these confounders and other random factors due to the small sample size, potentially leading to a faulty classification. This will need to be refuted in future studies, with a strict balance of such parameters between groups and a separate validation group to evaluate the neuromarker performance. Moreover, it is yet to be proven that the use of the BNA neuromarker for differentiation between the two non-demented subject groups relates to the diagnosis of PD and not to cognitive differences per se. The lack of comprehensive neurocognitive testing of enrolled subjects is a barrier to exploring this possibility in the present study. Furthermore, future studies will need to investigate whether the BNA neuromarker is specific for PD among other brain disorders, both degenerative and not. While the restriction of the BNA features to the cognitive domain may represent a limitation of the importance of this neuromarker for PD, we showed that it correlates with motor symptom severity, supporting its association with other PD related pathology. In addition, in the past several decades there have been a substantial research on abnormal oscillatory and synchrony patterns in the CBGTC loops in PD patients [70,71]. Specifically, excessive cortical beta band activity, beta synchronization within BG and synchronization between BG and cortex in PD patients have been repeatedly found (summarized in [70]). Adding such EEG features in combination with ERPs may boost performance of an early-stage neuromarker of PD.

Further studies assessing BNA’s potential for ESPD diagnosis should include large and diverse PD cohorts, such as patients in the premotor prodromal phase of the disease. Analysis of the BNA’s value in other brain disorders is recommended to identify diseases-specific fingerprints and to differentiate PD from atypical parkinsonian disorders. Additionally, longitudinal studies would help to identify novel BNA neuromarkers for disease progression or for progression of cognitive dysfunction in PD.

## Supporting information

S1 Fig Schematic illustration of the EEG/ERP tasks. (A) Auditory Oddball. (B) Visual Go/No-Go. ms, milliseconds.
(PDF)

S2 Fig Schematic depiction of BNA scores. (A) Dashed line shows the amplitude of the ERP peak in microvolts. (B) Dashed line shows the latency of the ERP peak from the stimulus onset (time zero), in milliseconds. (C) Horizontal location of STEP peak activity (Left-Right), arbitrary units (range -1 to 1). (D) Vertical location of STEP peak activity (Posterior-Anterior), arbitrary units (range -1 to 1). (E) Topographic similarity of the patient’s STEP in comparison with the STEP of the normative group, after alignment of the STEP peaks.
(TIF)

S1 Table Mean and SD of BNA scores of the five most important neuromarker features. (PDF)

S2 Table Scalp regions used for event related potential variability (ERPv) averaging. (PDF)

S3 Table ROC curve data. (PDF)

S4 Table Summarized physiological explanations of the Neuromarker features. (PDF)

S5 Table Demographic and clinical characteristics of the established PD group. (PDF)

## Abbreviations

AOB: auditory Oddball
BDI: Beck Depression Inventory
BG: basal ganglia
BNA: Brain Network Analytics
CBGTC: cortico-basal ganglia-thalamo-cortical
DMT: Disease-modifying therapy
EEG: electroencephalography
ERPs: event-related potentials
ERPv: ERP variability
ESPD: early-stage PD
fMRI: functional magnetic resonance imaging
FPR: False Positive Rate
GPi: globus pallidus pars interna
HC: healthy controls
LR: logistic regression
ML: machine learning
MMSE: mini-mental state exam
MoCA: Montreal Cognitive Assessment
mUPDRS: motor UPDRS
PD: Parkinson’s disease
ROC: receiver-operating characteristic
rTMS: repetitive transcranial magnetic stimulation
STEP: spatiotemporal parcel
UPDRS: Unified Parkinson’s Disease Rating Scale
VGNG: visual Go/No-Go
