# Supplementary figures and images for: Identification of an early-stage Parkinson’s disease neuromarker using event-related potentials, brain network analytics and machine-learning
Source: PLoS One. 2022 Jan 7;17(1):e0261947. doi: 10.1371/journal.pone.0261947 (PMC8741046; doi:10.1371/journal.pone.0261947)

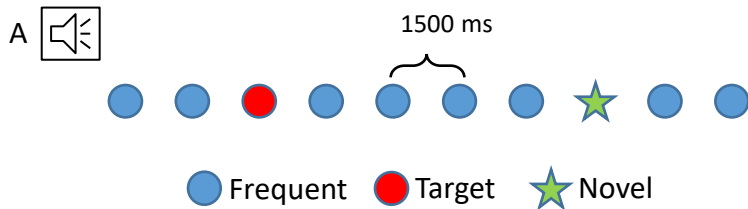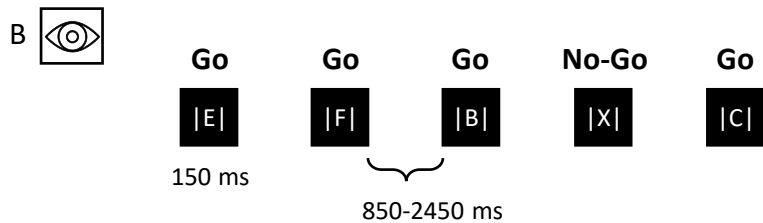

Supplement: S1 Fig — (A) Auditory Oddball. (B) Visual Go/No-Go. ms, milliseconds. (PDF) [file pone.0261947.s001.pdf]

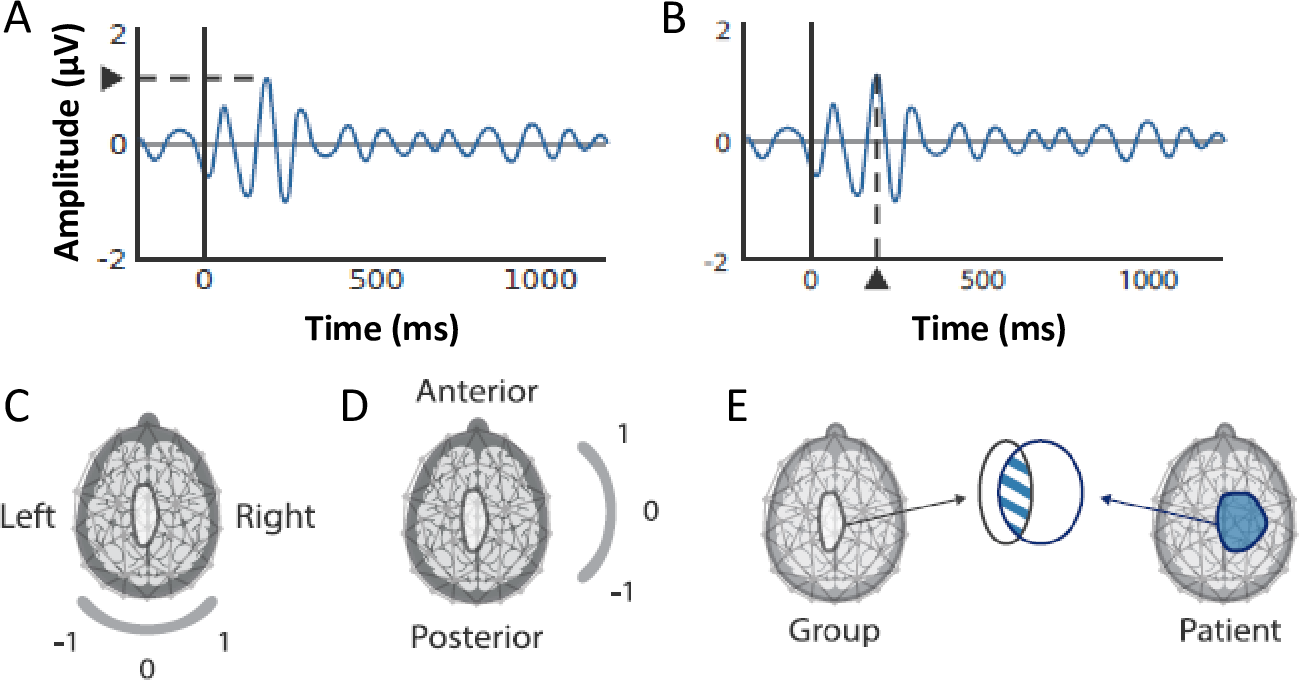

Supplement: S2 Fig — (A) Dashed line shows the amplitude of the ERP peak in microvolts. (B) Dashed line shows the latency of the ERP peak from the stimulus onset (time zero), in milliseconds. (C) Horizontal location of STEP peak activity (Left-Right), arbitrary units (range -1 to 1). (D) Vertical location of STEP peak activity (Posterior-Anterior), arbitrary units (range -1 to 1). (E) Topographic similarity of the patient’s STEP in comparison with the STEP of the normative group, after alignment of the STEP peaks. (TIF) [file pone.0261947.s002.tif]
