# Supplementary material for: Identification of an early-stage Parkinson’s disease neuromarker using event-related potentials, brain network analytics and machine-learning
Source: PLoS One. 2022 Jan 7;17(1):e0261947. doi: 10.1371/journal.pone.0261947 (PMC8741046; doi:10.1371/journal.pone.0261947)
Supplement: S1 Table — (PDF) [file pone.0261947.s003.pdf]

S1 Table. Mean and SD of BNA scores of the five most important neuromarker features

		Early PD		Healthy Control	
**Domain**	**Function**	**Mean**	**SD**	**Mean**	**SD**
Motor related	Response-locked activity (Go P-200, Topo)	0.67	0.20	0.81	0.13
Early sensory processing	Early sensory processing (Nogo P50, Amplitude)	2.00	0.45	1.43	0.65
	Early sensory processing (Novel N100, Latency)	126.82	19.60	113.89	11.15
Filtering of information	Filtering of information (Frequent P200, Amplitude)	1.37	0.46	1.92	0.74
	Filtering of information (Novel P200, Topo)	0.63	0.25	0.78	0.17

PD, Parkinson's disease; SD, standard deviation; Topo, topographic similarity.
