# Supplementary material for: Identification of an early-stage Parkinson’s disease neuromarker using event-related potentials, brain network analytics and machine-learning
Source: PLoS One. 2022 Jan 7;17(1):e0261947. doi: 10.1371/journal.pone.0261947 (PMC8741046; doi:10.1371/journal.pone.0261947)
Supplement: S2 Table — (PDF) [file pone.0261947.s004.pdf]

S2 Table. Scalp regions used for event related potential variability (ERPv) averaging

Area	Electrodes
Left Frontal	AF3, F5, F3
Medial Frontal	AFz, F1, Fz, F2
Right Frontal	AF4, F4, F6
Left Central	FC5, FC3, C5, C3, CP5, CP3
Medial Central	FC1, FCz, FC2, C1, Cz, C2, CP1, CPz, CP2
Right Central	FC4, FC6, C4, C6, CP4, CP6
Left Occipital Parietal	P7, P5, P3, PO7, PO3, O1
Medial Occipital Parietal	P1, Pz, P2, POz, Oz
Right Occipital Parietal	P4, P6, P8, PO4, PO8, O2
