# Supplementary material for: Identification of an early-stage Parkinson’s disease neuromarker using event-related potentials, brain network analytics and machine-learning
Source: PLoS One. 2022 Jan 7;17(1):e0261947. doi: 10.1371/journal.pone.0261947 (PMC8741046; doi:10.1371/journal.pone.0261947)
Supplement: S3 Table — (PDF) [file pone.0261947.s005.pdf]

S3 Table. ROC curve data

Cutoff point	Sensitivity	Specificity
0.32	0.53	0.90
0.33	0.58	0.90
0.35	0.63	0.90
0.35	0.63	0.87
0.35	0.68	0.87 *
0.39	0.68	0.83
0.40	0.68	0.80
0.48	0.68	0.77
0.49	0.68	0.73
0.53	0.74	0.73
0.60	0.74	0.70
0.69	0.74	0.67
0.75	0.74	0.63
0.76	0.74	0.60
0.78	0.74	0.57
0.81	0.74	0.53
0.86	0.74	0.50

Data points of the central portion of the ROC curve depicting the sensitivities and specificities at different cutoff points. The blue box designates the cutoff with the best sensitivity while maximizing specificity above chance level. Asterisk indicate the values of the blue circle in Fig 1C.
