# Supplementary material for: Identification of an early-stage Parkinson’s disease neuromarker using event-related potentials, brain network analytics and machine-learning
Source: PLoS One. 2022 Jan 7;17(1):e0261947. doi: 10.1371/journal.pone.0261947 (PMC8741046; doi:10.1371/journal.pone.0261947)
Supplement: S4 Table — (PDF) [file pone.0261947.s006.pdf]

S4 Table. Physiological explanations of the neuromarker features

Feature	ERP Physiological explanation
ERPv leftCentral (Response-locked) VGNG	ERPv reflects the variability of the single trials. It is provided for each of 9 scalp regions (S2_Table; e.g., left central, medial frontal etc.) as the average of the ERPv of the electrodes in that region.
ERPv leftCentral VGNG	
ERPv leftFrontal VGNG	
ERPv medialFrontal VGNG	
ERPv rightCentral (Response-locked) VGNG	
ERPv rightFrontal VGNG	
Novel P50 theta Latency AOB	The P50 ERP component reflects early sensory processing (Pratt, 2011)
No-Go P50 alpha Amplitude VGNG	
Novel N100 alpha Latency AOB	The N100 ERP component reflects early sensory processing (Pratt, 2011)
Frequent P200 alpha Amplitude AOB	The P200 ERP component reflects filtering of information
Frequent P200 alpha Topo AOB	
Novel P200 alpha Topo AOB	
Novel N2 alpha P-A AOB	The N2 ERP component reflects cognitive control and novelty processing (Folstein and Van Petten, 2008).
Go P300 delta P-A VGNG	The classic P300 ERP component as is often elicited by a rare target stimulus in an Oddball task, reflects engagement of attentional resources, stimulus evaluation and classification, working memory and response selection (Polich, 2007, 2012); Similarly, the Go-P300 in the Go/Nogo task reflects "target" processing in the sense it requires a response, including response preparation and execution (Barry et al., 2014).
Go P-200 alpha Topo (Response-locked) VGNG	A positivity appearing 200 ms prior to the motor response in the Go condition; since it is locked to the response, it may reflect activity related to motor execution.

References

Barry RJ, De Blasio FM, Borchard JP. Sequential processing in the equiprobable auditory Go/NoGo task: children vs. adults. Clinical Neurophysiology. 2014 Oct 1;125(10):1995-2006.

Folstein JR, Van Petten C. Influence of cognitive control and mismatch on the N2 component of the ERP: A review. Psychophysiology. 2008;45: 152–170. doi:10.1111/j.1469-8986.2007.00602.x

Polich J. Updating P300: An integrative theory of P3a and P3b. Clin Neurophysiol. 2007;118: 2128–2148. doi:10.1016/j.clinph.2007.04.019

Polich J. Neuropsychology of P300. In: Luck SJ, Kappenman ES, editors. The Oxford handbook of event-related potential components. Oxford University Press; 2012. pp. 159–188.

Pratt H. Sensory ERP Components. In: Luck SJ, Kappenman ES, editors. The Oxford Handbook of Event-Related Potential Components. Oxford university press; 2011. pp. 89–114.
