# Supplementary material for: Identification of an early-stage Parkinson’s disease neuromarker using event-related potentials, brain network analytics and machine-learning
Source: PLoS One. 2022 Jan 7;17(1):e0261947. doi: 10.1371/journal.pone.0261947 (PMC8741046; doi:10.1371/journal.pone.0261947)
Supplement: S5 Table — (PDF) [file pone.0261947.s007.pdf]

S5 Table. Demographic and clinical characteristics of the established PD group

Number of participants	20
Males, n (%)	15 (75)
Mean age, years (SD)	64.2 (8.4)
Mean duration of PD, years (SD)	6.7 (6.3)
Mean Hoehn and Yahr stage (SD)	2.2 (0.6)
Mean Beck Depression Inventory (BDI) score (SD)	6.9 (4.3)
Mean MMSE score (SD)	28.9 (1.4)
Mean mUPDRS score (SD)	31.8 (10.8)
Treatments for PD, number of patients (%)	
Levodopa	17 (85)
Monoamine oxidase inhibitor	18 (90)
Dopamine agonist	11 (55)
Amantadine	7 (35)
Anticholinergic agent	1 (5)

SD, standard deviation; BDI- Beck Depression Inventory; MMSE, mini mental status scale; mUPDRS, motor examination (part III) score of the Unified Parkinson's Disease Rating Scale.
